# Response to Commentary: Effect evaluation of *Sahtak bi Sahnak*, a Lebanese secondary school-based nutrition intervention: a cluster randomised trial

**DOI:** 10.3389/fnut.2024.1396571

**Published:** 2024-04-26

**Authors:** Liliane Said, Jessica S. Gubbels, Stef P. J. Kremers

**Affiliations:** ^1^Department of Nutrition and Food Sciences, Faculty of Arts and Sciences, Lebanese International University, Bekaa, Lebanon; ^2^Department of Health Promotion, NUTRIM School of Nutrition and Translational Research in Metabolism, Faculty of Health, Medicine, and Life Sciences, Maastricht University, Maastricht, Netherlands

**Keywords:** cluster randomized controlled trial, reproducibility, randomization, transparency, schools

In response to the commentary of Siddique et al. (1), some further clarifications are provided regarding the statistical analysis and description of the methods used. As the published paper focuses on the effect evaluation of the intervention rather than on describing the study protocol, we believe that the present response will add clarification to the article, especially for readers who may have a different perspective in approaching the analysis.

## 1 Randomization and sample size

In addition to the lines mentioned in “Study Design and Participants” in the original article, the randomization process and the sample size calculations are further elaborated in the text below:

### 1.1 Randomization

The target population in the current study included adolescents aged 15 to 18 years living in Beirut, Baalbeck, and Rayak. The Lebanese Ministry of Education and Higher Education (MEHE) divides secondary schools into two categories: (i) public schools and (ii) private schools. Each category is subdivided based on the location. Hence, secondary schools located in the target areas were selected and categorized as follows:

Next, schools from each cell (of the Table 1) were randomly chosen (by picking their name out of a basket) and contacted to obtain consent to participate. Once the school accepted to participate, it was randomly assigned to either intervention or control group with a toss of coin with 1:1 allocation.

**Table 1:** Sample of the created school lists.

		**Public**	**Private**
Rural	Baalbeck	School A1	School A'1
		School A2	School A'2
		School A3	School A'3
	Rayak	School B1	School B'1
Urban	Beirut	School C1	School C'1
		School C2	School C'2
		School C3	
		School C4	
		School C5	
		School C6	

In Baalbeck, there are only three public secondary schools and they all agreed to participate, so we had to recruit three private schools to balance the sample. Similarly, in Rayak, there is only one public secondary school, so in parallel one private school was allocated in this town. As for Beirut, we aimed to reach eight schools (i.e., four public and four private ones) to obtain the same number of secondary schools in the urban and rural study locations. However, only two private schools accepted to participate, so we recruited six public schools, instead of four. This justifies the adjustment for the type of school later in the statistical analyses.

All students within the same school were assigned to be part of the same study group (i.e., intervention vs. control) to avoid information contamination between groups as all students from the same school met in the same recreation ground during recess.

It is important to note that the randomization did not take into consideration the gender, BMI z-score, and grade/class distribution within schools. However, these variables were adjusted for later in the statistical analyses. In addition, and as was mentioned in the paper, it is potentially not possible to generalize the obtained results to the rest of the country or the rest of Lebanese adolescents as the current sample was not representative.

### 1.2 Sample size

According to the CONSORT 2010 updated guidelines for reporting parallel group randomized trials (2), authors should indicate the sample size calculation including the estimated outcomes, the α error level, the statistical power, and the standard deviation of the measurements for continuous outcomes.

The following equation was used to calculate *n* (3):

$\begin{array}{l} n=\frac{2{\left(Z_{\beta }+Z_{\alpha }\right)}^{2}\times \sigma^{2}}{\mu^{2}};\;taking\;into\;consideration: \end{array}$

- *Z*_β_ = 0.842 and β = 0.2.
- *Z*_α_=1.96 and α = 0.05.
- μ = 18.2% (4) representing the expected difference between the means of nutrition knowledge for the two groups being compared.
- σ = 0.54 (4) representing the standard deviation for the two groups.

This resulted in *n* = 139 per group, equalling *n* = 278 for both study groups. This method was similar to what other cluster randomized controlled trials (cRCT) adopted in their sample size calculation (5–7).

As the current study design is a cRCT, it is important to note that the coefficient of intracluster correlation (ICC) was not included in the calculation for the following reasons: (i) to our knowledge very few studies (8) focused on improving the dietary or nutrition knowledge among adolescents aged 15 to 18 years, and none were cRCT, hence evidence-based input for the ICC was lacking; (ii) second, we searched the literature for other cRCT including younger adolescents in Lebanon, but the only available study at that time did not indicate how the sample size was calculated, nor the ICC (9). The updated study published in 2020 did not indicate the details regarding the sample size nor the ICC value (10), which may reflect the lack of data regarding this issue (11); (iii) When looking at studies conducted in school settings among younger children, the ICC for children's nutritional knowledge was equal to 0 (12). Based on this, the calculated sample size remains the same as presented in the paper:

*n* = *n*_*I*_ [1+(*m*−1)*p*] (13);

- *n* is the required sample per arm.
- *n*_*I*_ is the required sample size per arm during an individually randomized trial.
- *p* is the ICC.
- *m* is the cluster size.

If ICC = 0, it means that: *n* = *n*_*I*._

As the current paper was mainly focused on the evaluation of the intervention outcomes rather than on describing the study protocol, we decided not to present these underlying details in the original paper to focus more on the outcomes and remain within the journal's word limits. We are happy to have the opportunity to provide these details in the current response.

## 2 Chi-square and *t-*test analysis

According to the CONSORT guidelines (14), a table showing baseline demographic characteristics for each study group should be included. Even though the randomization process prevents selection bias, it does not automatically assume that the study groups are equivalent at baseline (2). Since the number of schools in each study group was the same (eight schools in the intervention group and eight schools in the control group) and as the number of schools per location was also equal (eight schools from the urban region and eight schools from the rural one), our focus turned to evaluating the demographic characteristics at the individual level. This was further proved by the statistical tests conducted by the authors of the commentary as there was no significant difference in any variable of Table 1 related to demographics (1).

To evaluate the appropriateness of the randomization process related to the demographic characteristics of the sample, both Chi-square and independent *t*-test were performed. As was mentioned in the paper (see Statistical Analyses), Chi-square tests were used to analyse the differences in frequency of the categorical background characteristics (such as gender) between study groups, and the independent *t-*test was applied to compare the variation between study groups at baseline for continuous variables (such as the BMI *z*-score). The same tests were also used for these purposes in other cRCT (5, 15–18).

## 3 Clustering and nesting

The current study took into consideration the clustered design of the study by applying either the multilevel analysis or other tests as indicated below.

In the current statistical analysis, clustering included adolescents, schools, and school location. The grade or class (grades 10 and 11) was considered as an independent variable and not a cluster for the following reasons: (i) class size: the number of students per class in grade 10 is higher compared to grade 11. This means that the intervention may be more effective in smaller classes compared to larger ones; (ii) age category: to be able to evaluate the effectiveness of the intervention among older vs. younger adolescents later on. This explains the adjustment of the analysis for grade/class in Table 2 and the inclusion of this particle variable in the subgroup analysis in Table 3 (as indicated in the Statistical Analyses on page 5).

Concerning the other tests, some references state that there are cases that do not require running a multilevel analysis, and a multiple regression test could be used instead (19). For instance, when the random intercept is non-significant, it makes sense not to run the multilevel analysis, especially when the intercept does not vary from one school to another.

It is worth noting that after running the multilevel analyses for the unhealthy items score, we obtained a B value of (−1.40) with a 95% Confidence Interval of (−1.88) to (−0.93), and *p*-value of < 0.001. The direction, magnitude, and significance of the results were similar as with the multi-regression analyses.

## 4 Reproduction of statistical analysis

As reproducibility is the base of any scientific work, we thank the authors for their time in reproducing the statistical analyses. The authors reproduced the results based on the shared data, the codebook, and the described methodology in the paper, which suggests that the methodology related to running the tests is thoroughly and sufficiently described allowing for a sound reproduction of the results.

It is important to note that adolescents, schools, and school location were considered in the multilevel analysis, whereas the authors of the commentary have only relied on adolescents and schools. Concerning the reproduced tests in Table 1 of the commentary, the small differences related to the confidence of interval are due to adding one additional covariate (i.e., age) which was not considered in the initial analysis. As mentioned in the footnote of Table 2, all models were adjusted for gender, class/grade, type of school, school location, BMI *z*-score, and obtained scores at baseline. Age was not among them because we already included “class/grade” which takes into consideration older and younger adolescents.

As for the reproduced tests in Table 2 of the commentary, the significant differences in the B values of the total dietary knowledge score based on the baseline score are due to dividing the sample according to the healthy items score at baseline, and not according to the knowledge score at baseline (pre-intervention), which is not what was done in the analysis shown in the paper. This is indicated in the attached code (see “if healthy_score_category_pre = = 1”).

It is also important to note that some variables were counted more than once in the reproduced results of Siddique et al. (1). For instance, in the initial analysis shown in the paper, when dividing the sample into two groups: grade 10 and grade 11, the grade/class variable was not added to the analysis, as it was already taken into consideration. The same goes for weight status and knowledge score at baseline. In other words, when dividing the sample based on the weight status, there is no need to adjust the model for BMI *z*-score, and when dividing the sample based on the baseline knowledge score, there is no need to adjust for the baseline knowledge score once again.

Additionally, the significant differences in the healthy items adherence score based on the location (Table 3 of the commentary) are also due to adding the wrong variable. The dependent variable is the healthy items score post-intervention, and not the unhealthy items score post-intervention.

Concerning the revised tests, the same comments apply as mentioned above.

Finally, we would like to thank the authors for engaging in the discussion and appreciate the opportunity to provide further details with regard to our original paper. We believe Siddique and colleagues have presented a different approach for the data analysis, presenting the reader with a valuable alternative analytical approach to consider. In this response, we have provided a thorough explanation of why certain choices in our analytical approach were made. We feel it is important to stress that both approaches can be argued for, and both result in the same conclusion.

## Author contributions

LS: Writing – review & editing, Writing – original draft. JG: Writing – review & editing. SK: Writing – review & editing.
